# Sociodemographic and environmental characteristics associated with thoughts of death and suicidal ideation in community-dwelling residents of a rural town in Japan: analyses from a perspective of accompanying problems

**DOI:** 10.1186/s12889-024-18538-2

**Published:** 2024-04-23

**Authors:** Kozue Morikawa, Kyoko Nomura, Daisuke Onozawa, Hisanaga Sasaki, Yoshiki Morikawa

**Affiliations:** 1 Department of Environmental Health Science and Public Health, Akita University Graduate School of Medicine, 1-1-1 Hondo, Akita, 010-8543 Japan; 2 Department of Judo Physical Therapy, Teikyo Heisei University Faculty of Health Care and Medical Sports, Chiba, Japan; 3 Department of Health Science and Nursing, Akita University Graduate School of Medicine, Akita, Japan

**Keywords:** Health problems, Human relations problems, Financial problems, Suicidal ideation, Thoughts of death

## Abstract

**Objective:**

Suicide prevention has been focused on primary prevention as a group rather than individuals. However, we aimed to identify sociodemographic and environmental characteristics of individuals with suicidal thoughts among rural residents in Japan.

**Methods:**

In 2015, a cross-sectional home visit survey was conducted in a rural town in Akita Prefecture. A total of 1,844 residents aged ≥ 20 years (response rate, 65%) answered a self-administered questionnaire about suicidal thoughts in the past one month. Multivariate logistic regression analyses were used to investigate sociodemographic and environmental characteristics associated with suicidal thoughts in models with accompanying problems for human relations problems (HRP), health problems (HP), and financial problems (FP), or with no accompanying problems.

**Results:**

In total, 218 (men 9.4%, women 13.8%) had suicidal thoughts with accompanying problems for HRP (*n* = 104), HP (*n* = 112), and FP (*n* = 72). The risk characteristics were Kessler Psychological Distress Scale scores ≥ 9 in models with HRP, HP, and FP or with no accompanying problems; being a woman and current smoking with no accompanying problems; absence of a person for help in a model of FP; and absence of family member for help in a model of HRP or with no accompanying problems. The mitigating factor were being optimistic (a domain of resilience skills identified by factor analysis) in models of HRP, HP, and FP or with no accompanying problems; being aged 70–79 and being aged ≥ 80 in a model of HRP.

**Conclusions:**

Suicidal thoughts among rural residents in Japan were associated with multifactorial sociodemographic and environmental characteristics.

**Supplementary Information:**

The online version contains supplementary material available at 10.1186/s12889-024-18538-2.

## Introduction

According to the World Health Organization (2021), more than 700,000 people worldwide take their own lives annually, and suicide is one of the leading causes of death. Suicide has long-term consequences for those left behind. Japan ranked eighth in suicide mortality rate among the 38 Organisation for Economic Co-operation and Development (2020) countries and had the highest suicide rate among the seven major industrialized countries. In fact, suicide was the leading cause of death in 2021 among those aged 10–39 years [1].

According to the Ministry of Health, Labour, and Welfare [2], the most frequently cited problem for suicide is “health problems”, with depression being the most common underlying condition. The second most frequently cited problem is “financial problems”, followed by “family issues”, “work issues”, “sexual issues”, and “school issues”, all of which can be subsumed under “human relations”. Although there have been studies on mortality due to causative illness that have taken mental disorders into consideration, very few have attempted to identify sociodemographic characteristics associated with the problems for death and suicidal ideation. Suicide prevention has been focused on a group of people rather than individuals as primary prevention. There have been two decades since the enactment of the enactment of Basic Law on Suicide Countermeasures but the actual suicide rate in Japan has not been reduced yet. Therefore, taking an approach incorporating these accompanying problems may be useful as a new insight for suicide prevention strategy [3, 4]. For example, a person with suicidal ideation resulting from a financial crisis may not feel able to speak to anyone about their private problems, and thus may require a different approach from that used for a person with suicidal thoughts owing to health problems. Another example illustrates that if a person has human relationship problems in the workplace or community, intermediators of human resource personnel, public/occupational health practitioners may offer an environmental approach including a community structure/work system reform, a harassment/mental health education seminar, and the amended (rework) program to segregate an offender from harassed individuals. Identifying negative and positive sociodemographic and environmental characteristics may be directly reflected in interventions for individuals with particular concerns. Hence, the purpose of this study was to clarify the sociodemographic and environmental characteristics associated with suicidal thoughts according to the accompanying problems.

We investigated residents of a rural town surrounded by mountains and heavy snowfall in Akita Prefecture, northern Japan. Akita Prefecture, one of the most aged of Japan’s 47 prefectures, has had the highest suicide rate over two decades [5]. Such high risk of suicide may be caused by depressive symptoms that were provoked by the lowest average wage of workers in Japan [2], very few numbers of big companies or industries, decreased melatonin due to lack of sunlight, and reduced frequency of outings due to cold weather and snowfall. A previous Japanese study with 83,100 older adults demonstrated that relative deprivation, which measures the magnitude of the difference in income among individuals, increases the risk of individual depression [6]. Akita Prefecture in particular has a long history of actively promoting suicide prevention initiatives in Japan, and has contributed significantly to the enactment of the Basic Law on Suicide Prevention [7].

## Method

### Design and sample

This cross-sectional study was based on a mental health survey conducted in March 2015 among residents aged ≥ 20 years in a rural town in Akita Prefecture, northern Japan. At the time of the survey, there were 3,359 (48% aged ≥ 65 years) residents with 1,421 households in the town. The town has a vast area of 282.13 km2, but 64.8% of its area is covered by forests. The main industries are services (38%), followed by manufacturing services (14%) and construction (12%). In 2015, the suicide rate in this town was 59.8 (two men and zero women) per 100,000 population, which was higher than that (25.7 per 100,000 population; 79 women and 183 men) of Akita Prefecture [8]. For this reason, one of our research members (HS) had been continuously involved in cities, towns and villages of Akita, giving lectures about suicide prevention and health consultations for the residents. The first survey on suicidal ideation was conducted in this town in 2001. This was the third survey conducted in the town after the second survey in 2006, and therefore, the understanding and cooperation of the residents were excellent. Prior to the survey, the purpose of the investigation about suicidal thoughts was explained in the town's public newsletter. The health promoter volunteers distributed a self-administered questionnaire to all households in this town and the responses were obtained from 1,976 participants (response rate, 65%).

### Thoughts of death and suicidal ideation and accompanying problems

The participants were asked, “Have you had thoughts of dying or committing suicide in the past one month? (Yes/No/Sometimes)” Responses of “Sometimes” and “Yes” were considered indicative of thoughts of death and suicidal ideation. We also asked those who answered “Yes” or “sometimes” to have any problem regarding mental health problems, physical health problems, human relation within a family, general human relation, and financial problem. Respondents were allowed to provide multiple answers because these problems usually coexist. For example, even if initially caused by a relationship, it can lead to depression or other mental illness, making it impossible to work and resulting in financial hardship. We categorized the responses into three groups including “Human relations problems” including both family issue and general human relationship such as having an affair, bullying, harassment, and social withdrawal at school, in the community, or at the workplace, “Health problems” which covered both mental and physical health, and “Financial problems” which covered debt and financial crisis at the individual or community/society levels.

### Covariates

The survey items were gender; age; family structure; presence of suicide in the surroundings; psychological distress; resilience skills, including optimism, human resourcefulness, and problem-solving orientation. The presence of suicide in the surroundings was asked if you had someone who you know committed a suicide. If the respondent answered “yes”, we asked who the person was with three response choices (i.e., Family/Relatives, Friends/Acquaintance, Other).

Psychological distress was measured with the Kessler Psychological Distress Scale (K6, 9 or higher vs. 8 or lower) used to screen for mood and other disorders in community mental health epidemiological surveys [9]. Motohashi et al. [7] For resilience skills, we used a 21-item scale developed and validated by the Japan Society of Personality Psychology ( [10]; Appendix) and extracted three domains with nine items by principal factor analysis with Promax rotation (Supplementary Table 1): “Being optimistic”, “Human resourcefulness”, and “Problem-solving orientation”. The three domains of resilience skills were used as explanatory variables in the logistic regression model (described below).

### Statistical analyses

**Fig. 1:**
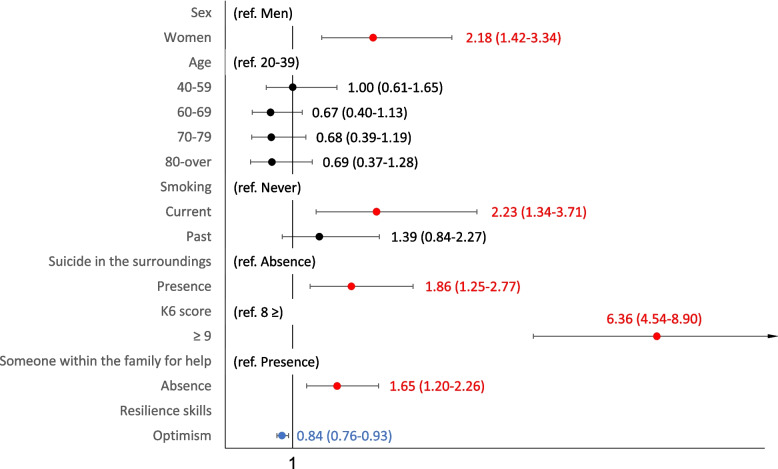
Sociodemographic characteristics of death and suicidal ideation (overall). Shows the overall results of the multivariate logistic regression analysis with multiple assignments for rarefaction and suicidal ideation

**Fig. 2:**
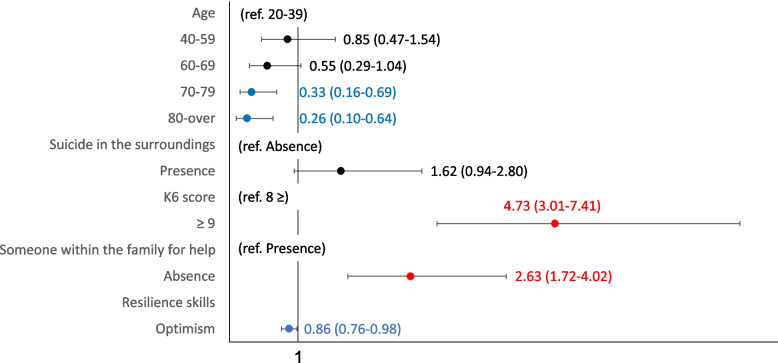
Sociodemographic characteristics of death and suicidal ideation (human relations problems). Shows the human relations problems of the multivariate logistic regression analysis with multiple assignments for rarefaction and suicidal ideation

**Fig. 3:**
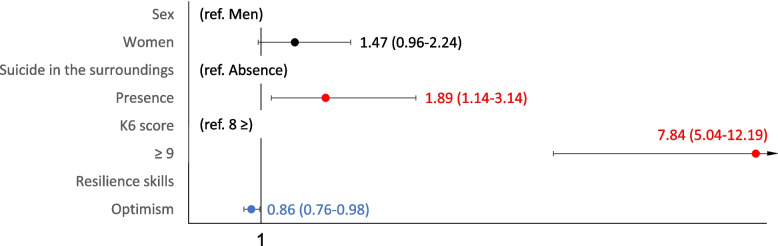
Sociodemographic characteristics of death and suicidal ideation (health problems). Shows the health problems of the multivariate logistic regression analysis with multiple assignments for rarefaction and suicidal ideation

**Fig. 4:**
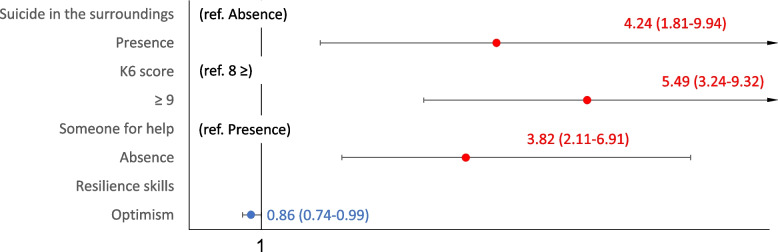
Sociodemographic characteristics of death and suicidal ideation (financial problems). Shows the financial problems of the multivariate logistic regression analysis with multiple assignments for rarefaction and suicidal ideation. Mitigating characteristics could not be extracted

Participant characteristics, including thoughts of death and suicidal ideation, accompanying problems, suicide in the surroundings, someone for help, and K6 scores, were compared between genders using a chi-square or t-test as appropriate. We investigated sociodemographic and environmental characteristics associated with thoughts of death and suicidal ideation during the past month, we used a logistic regression model (Fig. 1 Supplementary Table 2). Furthermore, we asked those about the accompanying problems with their thoughts, and we aimed to identify the socio-demographic and environmental characteristics for relationship problems (Fig. 2, Supplementary Table 3), health problems (Fig. 3, Supplementary Table 4) and economic deprivation (Fig. 4, Supplementary Table 5), respectively.

We estimated odds ratios (OR) for having thoughts of death and suicidal ideation, along with 95% confidence intervals (CI). We constructed multivariate models using a stepwise selection method and multiple imputation. Stepwise model selection automatically selects fewer predictor variables to build the best-performing logistic regression model. For multiple imputation, we first confirmed the missing data at random and then used multivariate imputation with the chained equations algorithm, creating 50 multiply imputed datasets. The results of the analysis of covariance were combined by averaging, and standard errors were adjusted to reflect both within- and between-imputation variability using Rubin’s rules [11].

Two-tailed tests were used to determine significance at the 5% level. All statistical analyses were performed using SAS version 9.4 (SAS Institute, Cary, North Carolina, USA).

### Ethical considerations

A public health nurse visited each household and obtained written informed consent from all participants. This study was approved by the concerned ethics committee (No. 1308).

## Results

After excluding missing values on suicidal ideation, the data of 1,844 participants were analyzed (women, *n* = 981, 55%). Table 1 shows the participant characteristics according to gender. More than 60% of participants of both genders were aged ≥ 60 years. Men were more likely than women to be current smokers (36.1% vs. 5.6%, respectively, *p* < 0.001) and daily drinkers (45.2% vs.7.4%, respectively, *p* < 0.001). Table 2 shows thoughts about death and suicidal ideation, problems for suicide, suicide in the surroundings, someone for help, and K6 scores. Thoughts about death and suicidal ideation in the past month were more common among women than men (13.8% vs. 9.4%, *p* = 0.004). Of the 218 participants with thoughts of death and suicidal ideation, 104 (48%) reported human relations problems, 112 (51%) reported health problems, and 72 (33%) reported financial problems. When we limited to single response, there were only 59 had health problem, 34 had human relation, and 19 had financial problems.

**Table 1:** Characteristics of study participants according to gender (*n* = 1844)

	Overall		Missing	Man (*n* = 812)		Woman (*n* = 981)		Missing	*p*
	*n*	%		*n*	%	*n*	%		
Sex			51						
Man	812	45.3		-	-	-	-		
Woman	981	54.7		-	-	-	-		
Age			19					61	0.272
20–39	215	11.8		104	12.9	108	11.1		
40–59	475	26.0		217	26.9	250	25.6		
60–69	471	25.8		217	26.9	245	25.1		
70–79	419	23.0		168	20.8	239	24.5		
≥ 80	245	13.4		101	12.5	134	13.7		
Family structure			41	807		976		80	0.105
Single	154	8.5		59	7.4	95	9.8		
One generation (couple only)	414	23.0		195	24.5	209	21.6		
Other (2nd generation)	1235	68.5		543	68.1	663	68.6		
Smoking			114					154	< .001
Current	344	19.9		288	36.1	50	5.6		
Past	403	23.3		318	39.9	73	8.2		
Never	983	56.8		191	24.0	770	86.2		
Drinking			72					112	< .001
Everyday	440	24.8		361	45.2	69	7.4		
3–5 times/ week	165	9.3		101	12.7	58	6.2		
1–5 times / month	256	14.5		93	11.7	159	17.0		
Never	911	51.4		243	30.5	648	69.4		
	Overall		missing	Man		Woman		missing	*p*
Resilience skills	mean	SD		mean	SD	mean	SD		
Optimism	7.1	1.6	141	7.0	1.7	7.1	1.5	133	0.090
Human resourcefulness	14.5	2.8	135	14.4	2.8	14.5	2.7	123	0.472
Problem solving oriented	9.8	2.2	140	9.8	2.2	9.8	2.1	132	0.622

**Table 2:** Thought of suicidal ideation, reasons, suicide in the surrounding, someone for help, and K6 (*n* = 1844)

	Overall		Missing	Men		Women		Missing	*p*
	*n*	%		*n*	%	*n*	%		
Thought of death during past one month			17					67	0.002
No	1653	90.5		748	92.9	860	88.5		
Yes	174	9.5		57	7.1	112	11.5		
Suicidal ideation during past one month			22					70	0.137
No	1672	91.8		751	92.8	877	90.9		
Yes	150	8.2		58	7.2	88	9.1		
Thought of death and Suicidal ideation (i.e., suicidal ideation)			0					51	0.004
No	1626	88.2		736	90.6	846	86.2		
Yes	218	11.8		76	9.4	135	13.8		
Accompanied reasons for suicidal ideation									
Human relation	104/218	48		34	-	66	-	51	0.020
Health problems	112/218	51		39	-	72	-	51	0.027
Financial problem	72/218	33		32	-	39	-	51	0.970
Suside in the surroudings			74					119	0.834
Absence	522	29.5		228	29.1	278	29.5		
Presence	1248	70.5		556	70.9	663	70.5		
Who is the person?									
Family / Relatives	509	40.8		208	25.6	285	29.1	51	0.105
Friends / Acquaintance	556	44.6		288	35.5	260	26.5	51	< .001
Other	339	27.2		131	16.1	199	20.3	51	0.026
Someone for help			0					51	< .001
Presence	1709	92.7		726	89.4	933	95.1		
Absence	135	7.3		86	10.6	48	4.9		
Someone within the family for help			0					51	0.003
Presence	1252	67.9		583	71.8	639	65.1		
Absence	592	32.1		229	28.2	342	34.9		
Someone outside of the family who can help			0					51	< .001
Presence	1041	56.5		372	45.8	651	66.4		
Absence	803	43.6		440	54.2	330	33.6		
K6 contineous									
mean, sd	5.0	4.6	173	4.6	4.6	5.4	4.6	210	< .001
K6 category			173					210	0.013
≥ 9	346	20.7		137	18.2	198	22.5		
8 ≥	1325	79.3		618	81.9	681	77.5		

The majority (70.5%) of the participants (*n* = 1,248) reported that they had someone who committed suicide in their surroundings. Regarding death by suicide in the surroundings, approximately 41% reported that the person was a family member or relative, and another 45% reported that the person was a friend or acquaintance. Women were more likely than men to have someone for help (95% vs. 89%, respectively, *p* < 0.001), but this person was less likely to be a family member as compared to the case with male participants (65% vs. 71%, respectively, *p* < 0.001).

**Fig. 5:**
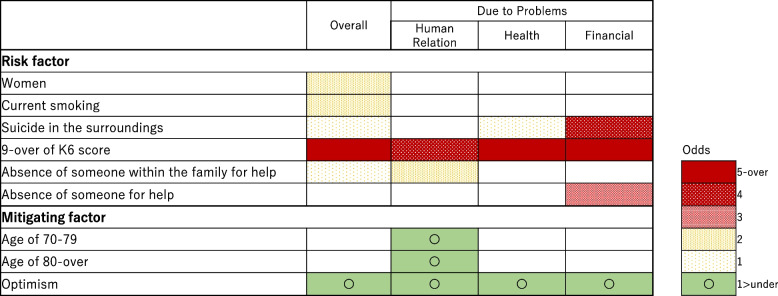
Heatmap of Sociodemographic characteristics of death and suicidal ideation. Shows a heatmap of the results of the multivariable stepwise logistic regression analyses with multiple imputation

Figure 5 shows a heatmap of the results of the multivariable stepwise logistic regression analysis with multiple imputation. Supplementary Table 2 shows the sociodemographic characteristics associated with thoughts about death and suicidal ideation in the past month. Multivariate models showed that the risk characteristics for suicidal thoughts, (with no accompanying problems), included being a woman, current smoking, suicide in the surroundings, K6 scores ≥ 9, absence of someone for help, and absence of someone within the family for help, while the coping characteristics were being optimism. Final model with multiple imputation that being a woman (OR 2.18, 95% CI: 1.42–3.34), current smoking (OR 2.23, 95% CI: 1.34–3.71), suicide in the surroundings (OR 1.86, 95% CI: 1.25–2.77), K6 scores ≥ 9 (OR 6.36, 95% CI: 4.54–8.90), and absence of someone within the family for help (OR 1.65, 95% CI: 1.20–2.26) were risk characteristics, while the coping factor was optimism (OR 0.84, 95% CI: 0.76–0.93).

The similar results with the accompanying problems for thoughts of death and suicidal ideation were shown in forest plot in Figs. 2, 3 and 4 and Supplementary Tables 3, 4 and 5 for human relations, health, and financial problems. The risk characteristics with human relations problems were K6 scores ≥ 9 (OR 4.73, 95% CI: 3.01–7.41), and absence of someone within the family for help (OR 2.63, 95% CI: 1.72–4.02), while the mitigating characteristics were being in their 70 s (OR 0.33, 95% CI: 0.16–0.69), being aged ≥ 80 years (OR 0.26, 95% CI: 0.10–0.64), and optimism (OR 0.86, 95% CI: 0.76–0.98). The risk characteristics with health problems were suicide in the surroundings (OR 1.89, 95% CI: 1.14–3.14), and K6 scores ≥ 9 (OR 7.84, 95% CI: 5.04–12.19), while mitigating characteristics was optimism (OR 0.86, 95% CI: 0.76–0.98). The characteristics with financial problems were suicide in the surroundings (OR 4.24, 95% CI: 1.81–9.94), K6 scores ≥ 9 (OR 5.49, 95% CI: 3.24–9.32), and absence of someone for help (OR 3. 82 95% CI: 2.11–6.91).

## Discussion

Investigating residents of a rural town in Akita Prefecture, Japan, we found that 11.8% of the participants had thoughts of death and experienced suicidal ideation in the past month. Adjusting for covariates, the risk characteristics identified were K6 scores ≥ 9 in models with accompanying problems of human relations, health, and finances or with no accompanying problems; being a woman and current smoking in a model with no accompanying problems; absence of a person for help in a model with financial problems; absence of family member for help in a model with human relations problems and with no accompanying problems. The mitigating characteristics were being optimistic (in models of human relations, health and financial problems or with no accompany problems), being aged 70–79 years (in models of human relations problems), and being aged ≥ 80 years (in models of human relations problems). These results are discussed in light of the findings of previous studies.

### Risk characteristics for thoughts of death and suicidal ideation

K6 score ≥ 9 is an independent and well-known risk characteristics for suicidal ideation [12]. In Japan, as stress and suicide have become social problems, the K6 was introduced in the 2007 National Health Survey [13] to assess mental health status. According to the 2007 report, the proportion of K6 scores ≥ 5 increased with age for both men and women: 22% of men and 27% of women aged ≥ 60 years [14]. In contrast, in our study, the proportion of those with scores ≥ 5 in their 60 s surpassed 59%. Thus, a higher prevalence of depressive symptoms in our sample might indicate that psychological distress perceived by residents in a countryside of Japan was stronger than those among general population, which made the K6 an independent risk factor for suicidal ideation in our study.

Being a woman was an independent risk factor for thoughts of death and suicidal ideation, in a model with no accompanying problems. Previously, it was shown that men are less likely to ask for help than women [15]. Our study demonstrated that although actual suicide rate was higher in men than in women in the town conducted in our study and Japan, suicidal thoughts was higher in women than in men [1], which is consistent with previous studies [16, 17]. Previous literatures reported that women have higher prevalence of psychiatric disorders, especially depression, anxiety and panic disorder, and borderline personality disorder [16, 17]. These conditions make women more likely to receive treatment, which may have an impact on the actual rate of suicide completion.

We did not ask for details about human relations problems because of the delicate nature of the issue. In addition, such sensitive issues cannot be resolved by third parties. Given that family support is a powerful tool to manage psychological stress in women [18], no support from the family is a sign of poor psychological outcomes. In such cases, public health professionals should actively introduce alternative community/workplace or neighborhood support [18] as well as drastic support for human relations.

Suicide in the surroundings was a risk factor, in models with health, and financial problems or with no accompanying problems. A systematic review reported that 20.3% of suicides are clustered and contagious [19]. A UK study reported that adults bereaved by suicide were more likely to attempt suicide than those bereaved by natural deaths [20]. One-third of the bereaved reported that they were likely to have died by suicide [21]. In the suicide contagion mechanism, bereaved family members are more prone to discrimination and prejudice against suicide in their surroundings. Eventually, they are easily left alone, feeling anxious and hopeless [22]. Compared to a situation involving the sudden death of a family member, suicide-bereaved families were more likely to blame themselves [23]. Indeed, in communities affected by suicide, the people left behind find it difficult to speak about the issue [24]. We believe that people who have lost someone to suicide need an outlet to express their sorrow, anger, and frustration and receive community-based support. For this reason, talking about suicide should not be taboo, and there is a need to build a community in which people can openly discuss the issue, resulting in decreased discrimination and prejudicial attitudes toward suicide [24].

The absence of someone for help was identified as a risk characteristics for suicidal ideation with financial problems and no accompanying problems. Financial issues are independent risk characteristics for depression and suicidal ideation/attempts [25]. Experience of economic hardship increases with financial threats [26], which may further increase the levels of suicidal ideation and confusion [27]. Previous studies agree that the presence of a person to help plays a protective role against psychological stress. A qualitative study that investigated men at risk of suicide in the face of employment, housing, and financial difficulties reported that the presence of someone for help enabled them to regain a sense of control over their lives [28]. Another study reported that the effects of economic stressors decrease in the presence of social support [29]. Thus, healthcare and welfare service providers should be aware that individuals with financial problems may be isolated, find it difficult to seek help, and also act upon the fact that thoughts of death and suicidal ideation may be alleviated by social support at the individual, workplace, and organizational levels.

Human relations problems may be private issues, including social withdrawal; bullying at the workplace or community; or intimate relationships that are not socially sanctioned. Our study did not ask for detailed personal information about each problem because solving sensitive issues may be complex for third parties. Nevertheless, neighbors, communities, family, and friends can support people facing problems by offering advice on coping strategies and medical information. A large body of scientific evidence shows that being surrounded by high-quality intimate relations and feeling socially connected are associated with a lower risk of all-cause mortality and various diseases [30].

Current smoking status was the only significant risk factor for suicidal ideation in a model with no accompanying problems. In our study, 26% of those with suicidal ideation were current smokers; this rate was significantly higher than among those who did not have such thoughts. According to nationally representative samples of Australian young people aged 13–17 years, 32% of those who had smoked in the past 30 days reported self-harm or suicidal thoughts in the past 12 months, compared to only 5% of those who had never smoked [31]. The relationship between depression and smoking has been extensively studied, and a causal inference has been argued; however, a consensus has not yet been reached [32]. Furthermore, the risk of death from suicide among current smokers has increased among female smokers [33]. In this regard, we did not observe any statistical interaction between current smoking and gender or K6 scores. As the smoking effect was not identified in the model of the accompanying problems, it is not easy to conclude. Alternatively, a previous study demonstrated that substance abuse including smoking is highly associated with psychological distress. This means that the effect of smoking might have been masked by adjusting for K6 in multivariable logistic regression models [34]. In addition, self-reporting of smoking may be less accurate due to various bias (recall bias, underreporting bias, etc.). Nevertheless, owing to clear evidence of the harmful effects of smoking, public health practitioners should recommend replacing smoking with healthier alternatives while educating people about the health risks associated with smoking.

### Mitigating characteristics for suicidal thoughts

Being optimistic was a mitigating factor for suicidal ideation with human relations, health, and financial problems or in a model with no accompanying problems. “Being optimistic” derived from factor analysis included two items: “I feel that I can usually manage anything” and “Even if I am not sure about something, I think I can manage it in the end”. A meta-analysis has shown that optimism is associated with reduced suicidal ideation [35]. Optimism is considered as traits that represent relatively stable personalities and tendencies that an individual possesses [36]. Previous research found that optimism was positively related to both emotion-focused and problem-focused coping, suggesting optimism fully mediated the relation between coping strategies and depressive symptoms [37]. A study revealed optimism can be enforced by using cognitive behavioral therapy (CBT) in major depressive disorders [38]. Although CBT is still limited to patients because, in general, an entire course of CBT requires face-to-face sessions for at least four weeks, with the advances in IT technology, a new inventory of soft applications may help unmet needs in this area.

Being aged 70–79 years and ≥ 80 years were a mitigating factor for suicidal ideation due to human relations problems. Compared to those in their 60 s or younger, people aged ≥ 70 years may find it much easier to survive because their children have grown up. Alternatively, they are mature enough to solve most human relationships and thus no longer need to face uncontrollable human relations.

### Strengths and limitations

Data on suicide in marginalized communities, specifically among older adults, are difficult to obtain and are valuable in the context of the accelerating population aging in Japan. To our knowledge, this study is the first to analyze suicidal ideation and its accompanying problems in human relations, health, and financial problems.

However, this study had some limitations that must be addressed. First, suicidal ideation is not equal to suicide attempts/completions, and thus, our result may be limited to apply to those who had attempted suicide. For the comparison of the accompanying reasons between actual suicide and suicidal ideation in our study, we referred to suicide statistics issued by Akita Police Station Headquarters in 2014–2015 when the study was conducted (https://www.police.pref.akita.lg.jp/kenkei/statistics/suicide). Among 320 consecutive counts of suicide motives among those who committed suicide, 111 (34.6%) had health problems, 108 were unknown, 51 (15.9%) had other human relations, including family, school, and intimacy issues, and 40 (12.5%) had financial problems. In contrast, our study found that among those who had thoughts of death and suicidal ideation, health problems (*n* = 112, 51%) and human relations problems (*n* = 104, 48%) were the two top followed by financial problems (*n* = 72, 33%). Because the source population differs between our study, a rural town of one prefecture, and the whole prefecture, it is not easy to compare the exact numbers. Nevertheless, we are still able to understand that the impact of health problems is the most frequent problem in both populations of those who committed suicide and those who had not yet attempted but had suicidal thoughts.

Second, because of accompanying problems with thoughts of death and suicidal ideation, we asked participants to report multiple problems because these problems may usually coexist. As a result, our sub-analyses according to each problem may be similar because the same person contributes to more than one logistic model. Third, the setting was a single town in Akita Prefecture, the findings have limited generalizability. The geographic environment must also be considered, as the setting of this study is a rural town with a high aging population. A study in Australia reported that older rural individuals might be at a higher risk of suicide than their urban-dwelling counterparts owing to differences in employment, economy, and living conditions [39]. Nevertheless, as the participation rate surpassed 65%, our data are representative of a rural town in Japan; in fact, the 6.5% prevalence of K6 scores ≥ 13 was similar to that in the same northeastern region in 2006 [40]. In addition, considering that all regions in Japan, with the exception of the capital, will be aging in the future, the results of this study, conducted in Akita Prefecture, the most aged prefecture in Japan, can be widely applied to the elderly population. Fourth, our data were based on self-report, and lifestyles, including alcohol consumption and smoking, may be inaccurate or underreported. Fifth, we combined psychological and physical problems as one category of health problems, and human relations owing to school, family, or outside family for statistical purposes as one category of human relations. If we break down these problems, we were not able to perform multivariable analyses due to small numbers of each problem. Sixth, owing to the cross-sectional design, it may be difficult to draw causal inferences [16, 17].

### Practical implications

Mentally ill status requires medical evaluation, which allows for early intervention in suicidal ideation. Suicide in the surroundings may drive instant solution for those with health and financial problems. In addition, for those with financial problems, if the person does not have anyone for help, it could induce suicidal thoughts. Thus, consultation service for anyone who has private concerns could be a strategic intervention. If a person does not have anyone for help within their families, it also could induce suicidal thoughts generally or among those with human relations problems. Hence, being alone despite having a family does not guarantee a safety net. Thus, an individual approach using consultation services for human relations problems could also be helpful. Women are generally vulnerable but, actual suicide rate was much higher in men than in women [1], which requires careful interpretation of the present study. Because we only investigated suicidal ideation, not actual suicidal attempts/completions, special attention should be paid to this study.

In problem-specific suicide prevention, future studies are warranted if the individual prevention approach in the community is helpful in mitigating suicidal thoughts or the actual rate of suicide attempts. In addition, with the advance of information technology, coping strategies, including optimism, should be included in CBT, and the effectiveness of the new inventory must be accumulated with scientific evidence. In Japan, it has been almost two decades since the enactment of the Basic Law on Suicide Countermeasures. Unfortunately, the suicide rate is still one of the top public health agendas in Japan. Every effort should be made to reduce the number of suicides.

## Conclusion

We found that the sociodemographic and environmental characteristics were associated with suicidal ideation and identified several characteristics according to the accompanying problems for human relations, health, and financial problems. In the long history of suicide prevention in Japan, primary prevention has focused on groups rather than individuals. However, our results suggest that by paying attention to individuals' sociodemographic and environmental characteristics, we may be able to provide a prompt approach to high-risk individuals and support them by listening to their problems and providing some advice on coping strategies or medical support. Furthermore, these efforts may be enforced with the incorporation of community in the neighborhood.

Future research needs to step in with interventions in psychotherapy and educational programs, including seeking help or consultation and investigating how bereaved families or community residents who experience suicide in their surroundings are physically and mentally affected.

### Supplementary Information

**Supplementary Material 1.**

**Supplementary Material 2.**

**Supplementary Material 3.**

**Supplementary Material 4.**

**Supplementary Material 5.**

**Supplementary Material 6.**

## Data Availability

The datasets used and/or analyzed during the current study available from the corresponding author on reasonable request.
