# Supplementary material for: Sociodemographic and environmental characteristics associated with thoughts of death and suicidal ideation in community-dwelling residents of a rural town in Japan: analyses from a perspective of accompanying problems
Source: BMC Public Health. 2024 Apr 23;24:1130. doi: 10.1186/s12889-024-18538-2 (PMC11040995; doi:10.1186/s12889-024-18538-2)
Supplement: Supplementary file 1 — Supplementary Material 1. [file 12889_2024_18538_MOESM1_ESM.docx]

**Appendix**

1. I consider myself a tenacious person.
2. I am good at getting to know people on my own.
3. I have a wide range of friendships and am sociable.
4. I am willing to talk to people when I have misunderstandings with them.
5. I feel that I can usually manage anything.
6. I have a good understanding of my own personality.
7. I am good at reading people’s feelings and subtle changes in facial expressions.
8. I understand how a bad event affects my feelings.
9. I am able to persevere even when things are difficult.
10. I can control my emotions even when I have a bad day.
11. When I have a bad experience, I gather information to solve the problem.
12. I am relatively good at understanding other people’s ways of thinking.
13. Even if I am unsure about something, I think I can ultimately manage it.
14. I have always been good at relating to others.
15. I value hard work.
16. I am compassionate in my dealings with others.
17. I think I can manage to get through difficult events.
18. I can make a decision and stick with it to the end.
19. When I experience a bad event, I look for what I can gain from my current experience. (bad event)
20. I am often unsure of my thoughts and feelings that I understand well about my personality.
21. I am a physically strong person.
